# Supplementary material for: A conserved enzyme of smut fungi facilitates cell-to-cell extension in the plant bundle sheath
Source: Nat Commun. 2022 Oct 12;13:6003. doi: 10.1038/s41467-022-33815-7 (PMC9556619; doi:10.1038/s41467-022-33815-7)
Supplement: Supplementary file 1 — Supplementary Information [file 41467_2022_33815_MOESM1_ESM.pdf]

Supplementary Information:

A conserved enzyme of smut fungi facilitates cell-to-cell extension in the plant bundle sheath

Bilal Ökmen[1,2*], Elaine Jaeger[1], Lena Schilling[3], Natalie Finke[1], Amy Klemd[3], Yoon Joo Lee[1], Raphael Wemhöner[1], Markus Pauly[4], Ulla Neumann[5], Gunther Doehlemann[1*]

[1] *Institute for Plant Sciences, University of Cologne, BioCenter, Zuelpicher Str. 47a, 50674 Cologne, Germany*
[2] *Department of Microbial Interactions, IMIT/ZMBP, University of Tübingen, Tübingen, Germany*
[3]*Max-Planck-Institute for Terrestrial Microbiology, 35043 Marburg, Germany*
[4]*Institute for Plant Cell Biology and Biotechnology, Heinrich Heine University of Düsseldorf, Universitätsstr. 1, D-40225 Düsseldorf, Germany*
[5]*Central Microscopy, Max-Planck-Institute for Plant Breeding Research, 50829 Cologne, Germany*

Corresponding authors:

Gunther Doehlemann, e-mail: g.doehlemann@uni-koeln.de

Bilal Ökmen, e-mail: bilal.oekmen@zmbp.uni-tuebingen.de

Supplementary Figures:

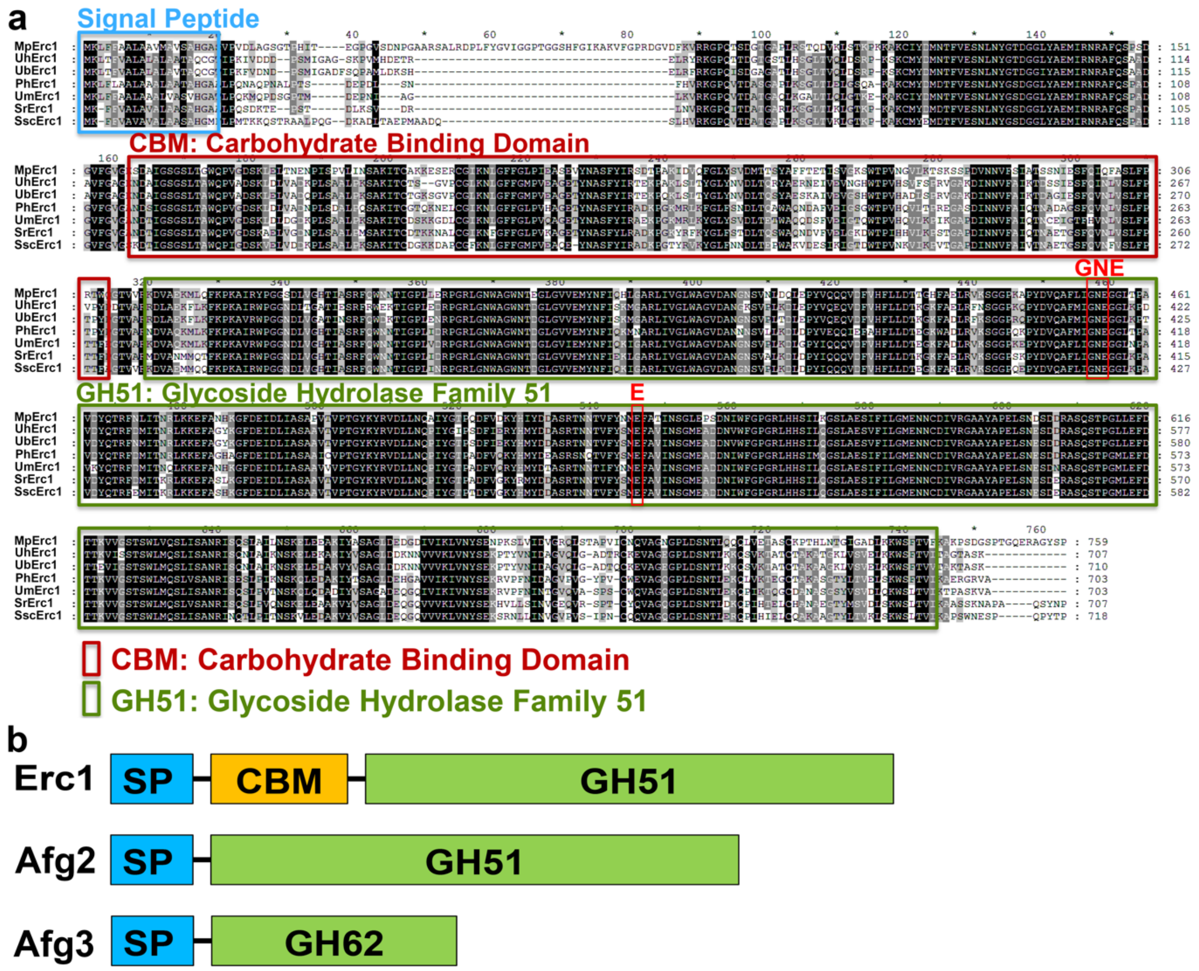

Supplementary Fig. 1. Amino acid alignment of Erc1 from different smut fungi. (a) ClustalOmega and GeneDoc software programs were used to construct this alignment. The blue box represents the predicted signal peptide using SignalP5, the red box shows the predicted carbohydrate-binding domain (CBM) and the green box shows glycoside hydrolase family 51 (GH51) domain. The GNE and E, which are depicted above the amino acid sequence in red, are predicted active sites for Erc1. Mp: *Melanopsichium pennsylvanicum,* Uh: *Ustilago hordei*, Ub: *Ustilago bromivora*, Ph: *Pseudozyma hubeiensis*, Um: *Ustilago maydis* Sr: *Sporisorium reilianum,* Ssc: *Sporisorium scitamineum.* **(b) Schematic presentation of Erc1 (Afg1), Afg2 and Afg3.** SP: Signal peptide; CBM: Carbohydrate-binding module, GH: Glycoside hydrolase family.

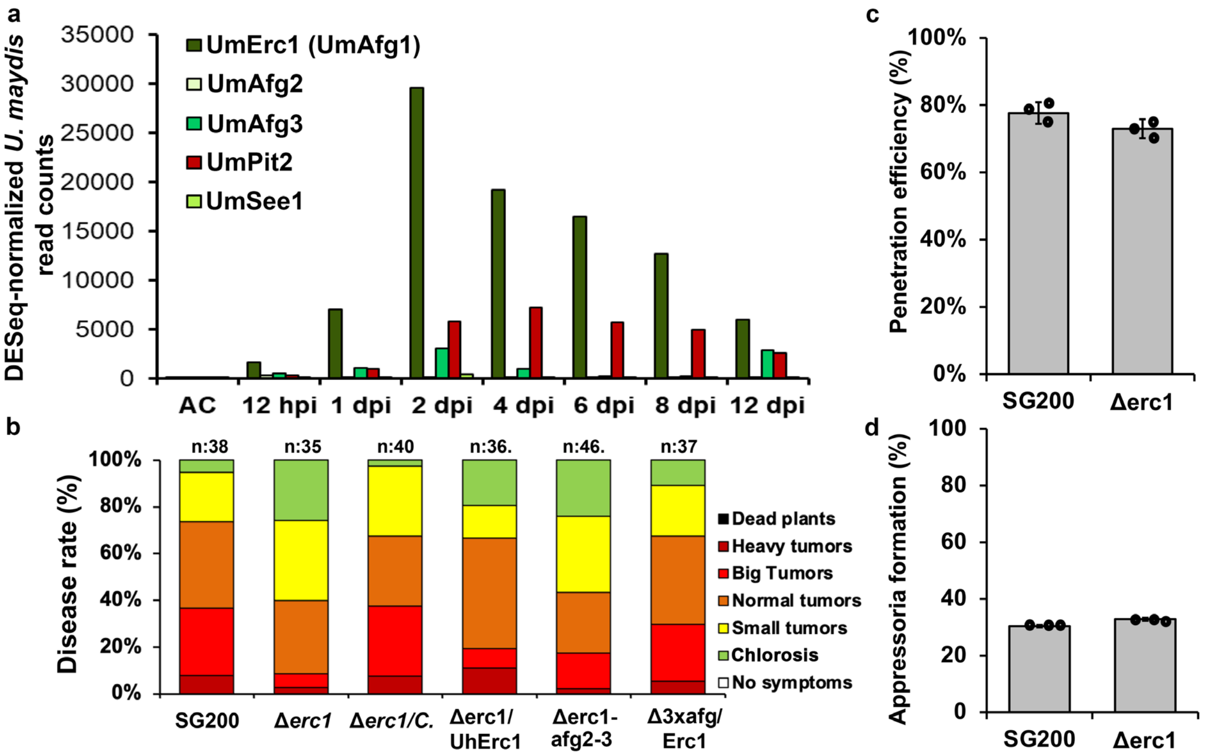

Supplementary Fig. 2. (a) Expression pattern of *GH51* genes, including *Erc1* (*Afg1*), *Afg2* and *Afg3*, in *Ustilago maydis* during maize infection. The transcriptome data created by Lanver *et al*. (2018) were used to create this bar graph (Lanver *et al.*, 2018). *UmPit2* (as a high expressed effector) and *UmSee1* (as a low expressed effector) effector genes were also added to the graph for reference. **(b)** Afg2 and Afg3 are not required for full virulence of *U. maydis* during maize infection. Disease symptoms caused by *Ustilago maydis* SG200, SG200Δerc1, SG200Δerc1/UhErc1, SG200Δafg2-3 and SG200Δ3xafg/Erc1 on Early Golden Bantam (EGB) maize cultivar at 12 days post inoculation (dpi). Disease rates are given as a percentage of the total number of infected plants. n: indicates total number of infected maize seedlings used in two independent biological replicates. **(c)** Quantification of penetration efficiency and **(d)** appressoria formation of *Ustilago maydis* SG200 and SG200Δerc1 strains on epidermis. Bar charts show data from three independent biological experiments.

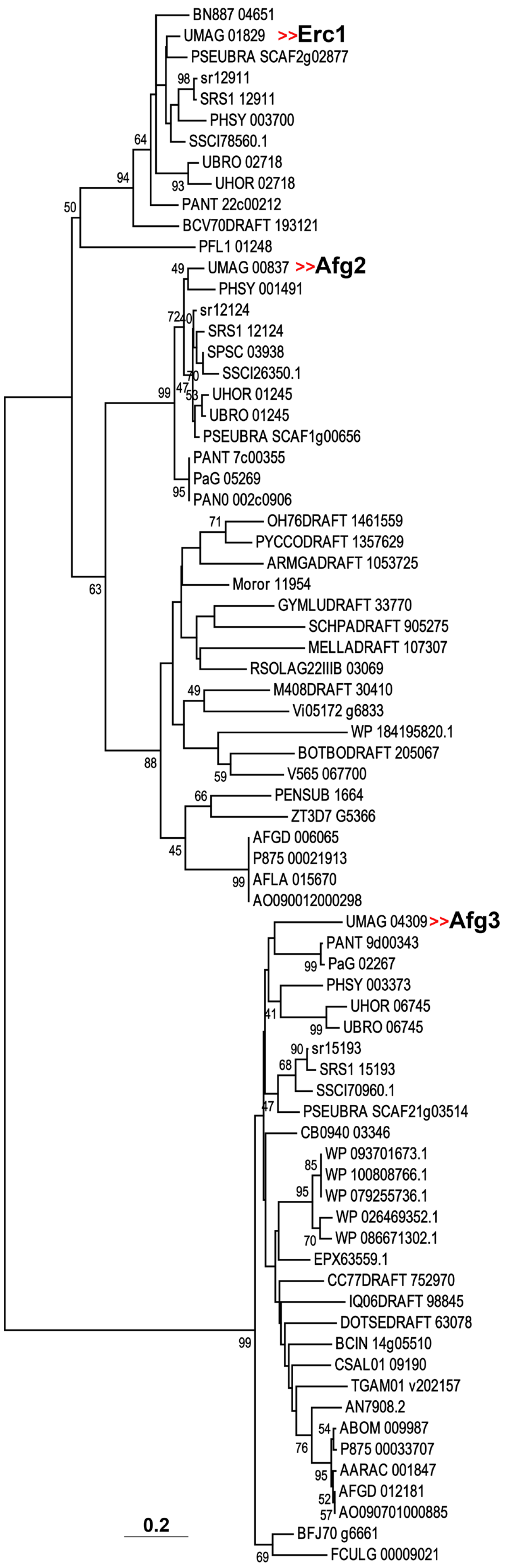
BN887 04651
UMAG 01829 >>Erc1
PSEUBRA SCAF2g02877
sr12911
SRS1 12911
PHSY 003700
SSCI78560.1
UBRO 02718
UHOR 02718
PANT 22c00212
BCV70DRAFT 193121
PFL1 01248
UMAG 00837 >>Afg2
PHSY 001491
sr12124
SRS1 12124
SPSC 03938
SSCI26350.1
UHOR 01245
UBRO 01245
PSEUBRA SCAF1g00656
PANT 7c00355
PaG 05269
PAN0 002c0906
OH76DRAFT 1461559
PYCCODRAFT 1357629
ARMGADRAFT 1053725
Moror 11954
GYMLUDRAFT 33770
SCHPADRAFT 905275
MELLADRAFT 107307
RSOLAG22IIIB 03069
M408DRAFT 30410
Vi05172 g6833
WP 184195820.1
BOTBODRAFT 205067
V565 067700
PENSUB 1664
ZT3D7 G5366
AFGD 006065
P875 00021913
AFLA 015670
AO090012000298
UMAG 04309>>Afg3
PANT 9d00343
PaG 02267
PHSY 003373
UHOR 06745
UBRO 06745
sr15193
SRS1 15193
SSCI70960.1
PSEUBRA SCAF21g03514
CB0940 03346
WP 093701673.1
WP 100808766.1
WP 079255736.1
WP 026469352.1
WP 086671302.1
EPX63559.1
CC77DRAFT 752970
IQ06DRAFT 98845
DOTSEDRAFT 63078
BCIN 14g05510
CSAL01 09190
TGAM01 v202157
AN7908.2
ABOM 009987
P875 00033707
AARAC 001847
AFGD 012181
AO090701000885
BFJ70 g6661
FCULG 00009021
0.2

Supplementary Fig. 3. Phylogenetic tree analyses of GH51 proteins. A minimum evolution tree was constructed by using an alignment of the full-length amino acid sequence of Erc1 homologs obtained from the NCBI database for different microorganisms. The minimum evolution tree was constructed by using the software Mega7 by using the minimum evolution algorithm performing 1000 bootstraps. PHSY_003700: *Pseudozyma hubeiensis* SY62; SRS1_12911: *Sporisorium reilianum* f. sp. *reilianum*; sr12911: *Sporisorium reilianum* SRZ2; SSCI78560.1: *Sporisorium scitamineum*; PANT_22c00212: *Moesziomyces antarcticus* T-34; PSEUBRA_SCAF2g02877: *Kalmanozyma brasiliensis* GHG001; UBRO_02718: *Ustilago bromivora*; UHOR_02718: *Ustilago hordei*; BN887_04651: *Melanopsichium pennsylvanicum*; BCV70DRAFT_193121: *Testicularia cyperi*; PFL1_01248: *Anthracocystis flocculosa* PF-1; PHSY_001491: *Pseudozyma hubeiensis* SY62; PaG_05269: *Moesziomyces aphidis* DSM 70725; Moror_11954: *Moniliophthora roreri* MCA 2997; PYCCODRAFT_1357629: *Trametes coccinea* BRFM310; BOTBODRAFT_205067: *Botryobasidium botryosum* FD-172 SS1; GYMLUDRAFT_33770: *Gymnopus luxurians* FD-317 M1; RSOLAG22IIIB_03069: *Rhizoctonia solani*; ARMGADRAFT_1053725: *Armillaria gallica*; SCHPADRAFT_905275: *Schizopora paradoxa*; AFLA_015670: *Aspergillus flavus*; AFGD_006065: *Aspergillus flavus*; AO090012000298: *Aspergillus oryzae*; P875_00021913: *Aspergillus parasiticus* SU-1; M408DRAFT_30410: *Serendipita vermifera* MAFF 305830; Vi05172_g6833: *Venturia inaequalis*; OH76DRAFT_1461559: *Polyporus brumalis*; PENSUB_1664: *Penicillium subrubescens*; ZT3D7_G5366: *Zymoseptoria tritici* ST99CH_3D7; V565_067700: *Rhizoctonia solani* 123E; PSEUBRA_SCAF1g00656: *Kalmanozyma brasiliensis* GHG001; PAN0_002c0906: *Moesziomyces antarcticus*; SRS1_12124: *Sporisorium reilianum* f. sp. *reilianum*; sr12124: *Sporisorium reilianum* SRZ2; PANT_7c00355: *Moesziomyces antarcticus* T-34; UHOR_01245: *Ustilago hordei*; SPSC_03938: *Sporisorium scitamineum*; UBRO_01245: *Ustilago bromivora*; SSCI26350.1: *Sporisorium scitamineum*; MELLADRAFT_107307: *Melampsora larici-populina* 98AG31; SSCI70960.1: *Sporisorium scitamineum*; sr15193: *Sporisorium reilianum* SRZ2; SRS1_15193: *Sporisorium reilianum* f. sp. *reilianum*; PANT_9d00343: *Moesziomyces antarcticus* T-34; PaG_02267: *Moesziomyces aphidis* DSM 70725; PSEUBRA_SCAF21g03514: *Kalmanozyma brasiliensis* GHG001; UBRO_06745: *Ustilago bromivora*; PHSY_003373: *Pseudozyma hubeiensis* SY62; UHOR_06745: *Ustilago hordei*; WP_086671302.1: *Amycolatopsis pretoriensis*; WP_100808766.1: *Streptomyces* sp. M56; WP_093701673.1: *Streptomyces* sp. MnatMP-M27; WP_079255736.1: *Streptomyces* autolyticus; WP_026469352.1: *Amycolatopsis balhimycina*; EPX63559.1: *Cystobacter fuscus* DSM 2262; WP_184195820.1: *Armatimonas rosea*; AARAC_001847: *Aspergillus arachidicola*; ABOM_009987: *Aspergillus bombycis*; AO090701000885: *Aspergillus oryzae* RIB40; BCIN_14g05510: *Botrytis cinerea* B05.10; P875_00033707: *Aspergillus parasiticus* SU-1; AFGD_012181: *Aspergillus flavus*; AN7908.2: *Aspergillus nidulans* FGSC A4; TGAM01_v202157: *Trichoderma gamsii*; CSAL01_09190: *Colletotrichum salicis*; BFJ70_g6661: *Fusarium oxysporum*; FCULG_00009021: *Fusarium culmorum*; DOTSEDRAFT_63078: *Dothistroma septosporum* NZE10; CB0940_03346: *Cercospora beticola*; IQ06DRAFT_98845: *Stagonospora* sp. SRC1lsM3a; CC77DRAFT_752970: *Alternaria alternata*

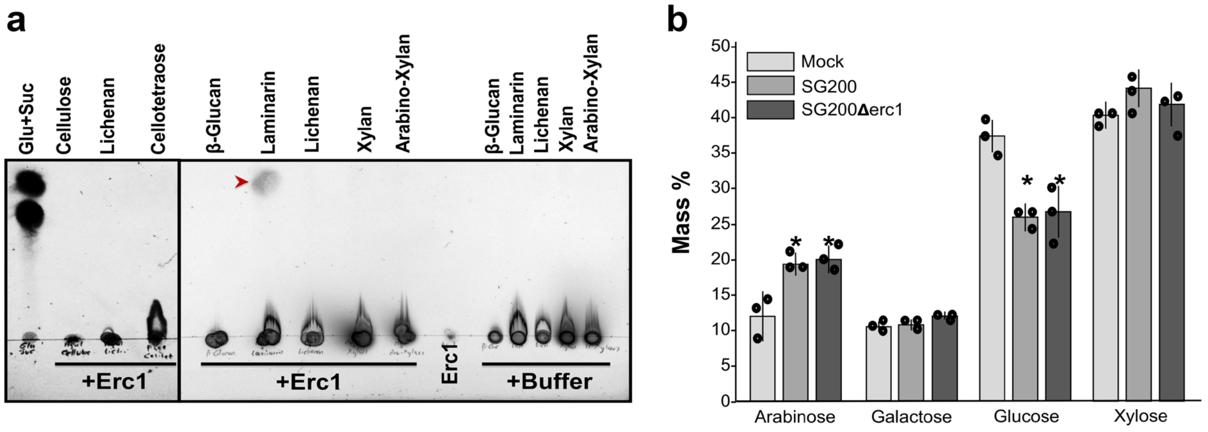

Supplementary Fig. 4. (a) Thin-layer chromatography (TLC) assay to show enzymatic activity of Erc1 on different polysaccharides, including cellulose, lichenan, cellotetraose, β-glucan, laminarin, xylan and arabinoxylan. A mixture of n-propanol:ethanol:water (7:2:1/v:v:v) was used as a mobile phase. Carbohydrates and their hydrolysis products were visualized by spraying the TLC plate with detection solution and subsequent drying at 100°C for approximately 15 min. Red arrow head indicates hydrolysis product of laminarin. Glucose + sucrose mix was used as reference. Similar results were observed at least in three independent biological experiments. **(b) Monosaccharide composition of maize cell walls.** Plant cell walls were isolated from mock, SG200 and SG200Δerc1-treated EGB maize leaves. The isolated plant cell walls were analyzed for their arabinose, galactose, glucose and xylose content. Data were presented as mean value ± SD of three independent biological experiments. Asterisks above bars indicate significant differences (p<0.05, two-tailed student's t-test). Calculated p-values are presented in Source Data.

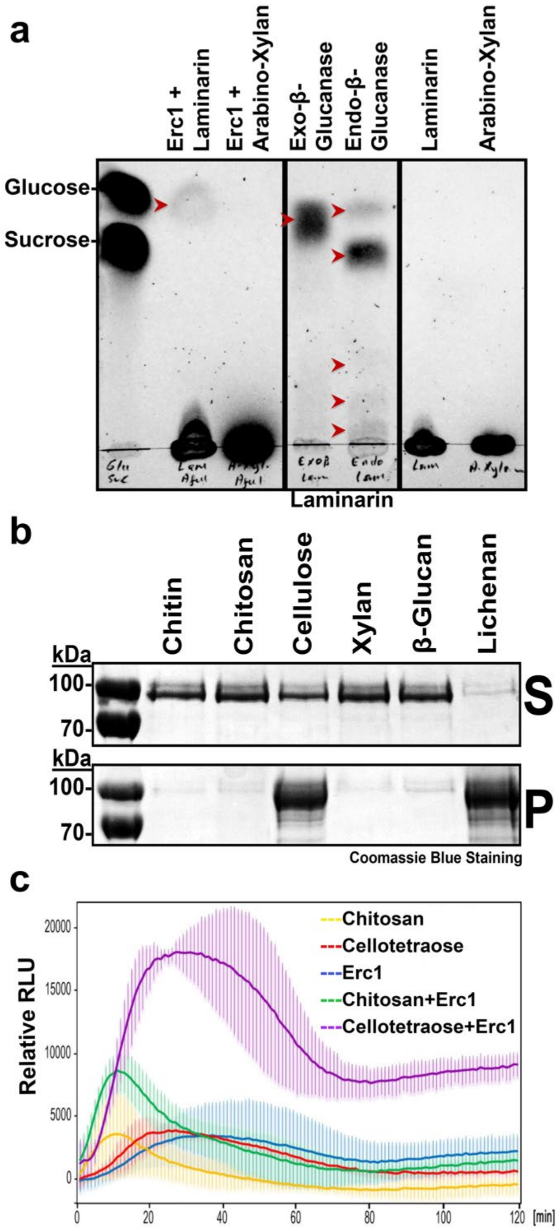

Supplementary Fig. 5. (a) Thin-layer chromatography (TLC) assay was performed to demonstrate exo-β-1,3-glucanase activity of Erc1 on laminarin. Glucose + sucrose mix was used as reference. A mixture of n-propanol:ethanol:water (7:2:1/v:v:v) was used as a mobile phase. Laminarin and its hydrolysis products were visualized by spraying the TLC plate with detection solution and subsequent drying at 100°C for approximately 15 min. Red arrow heads indicate hydrolysis products of laminarin. Commercial exo- and endo-β-1,3-glucanases were used as controls. Similar results were observed in two independent biological experiments. **(b)** Carbohydrate binding assay for recombinant Erc1 protein. Erc1 protein was incubated with insoluble chitin, chitosan, cellulose, xylan, β-glucan and lichenan. Subsequently, supernatant and pellet phases were analysed for presence of Erc1 protein via SDS-PAGE followed by coomassie blue staining. S: supernatant, P: pellet. Similar results were observed in three independent biological experiments. **(c)** To check whether Erc1 sequesters cellotetraose in order to prevent its recognition as a DAMP, a ROS-burst assay was performed with barley leaf disks incubated with cellotetraose, Erc1 and cellotetraose incubated with Erc1 recombinant protein. Chitosan was used as a negative control. Relative luminescence units (RLU) indicate ROS burst activity of treated barley leaf discs. The RLU are normalized with the buffer control. Data were presented as mean value ± SD of three independent biological experiments.

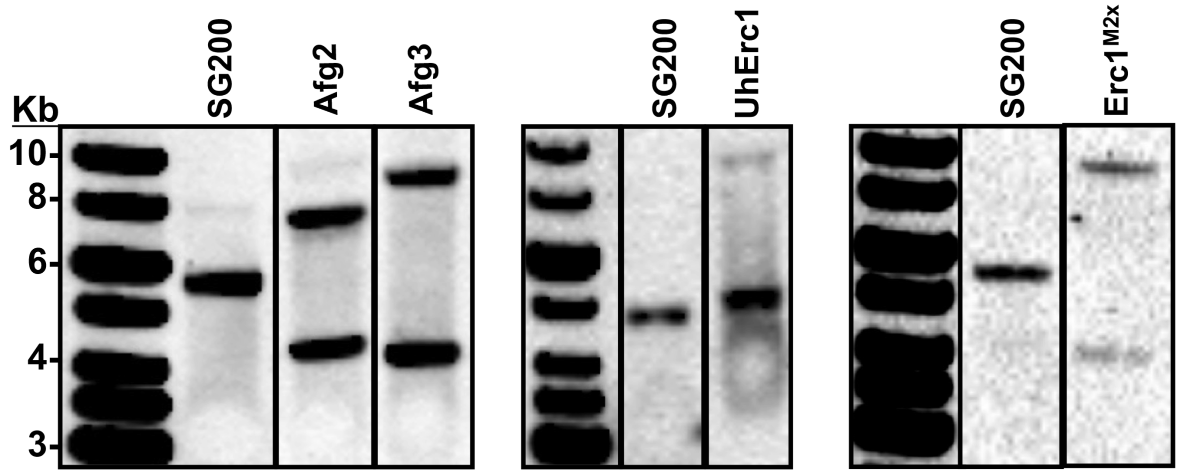

Cbx integration	Afg2 (*Bam*HI)	Afg3 (*Bam*HI)	UhErc1 (*Sac*I)	UhErc1^{M2x} (*Bam*HI)
Wild type:	5662 bp	5662 bp	4756 bp	5662 bp
Single integration:	4065-6534 bp	4061-8134 bp	3921-6949 bp	4073-9352 bp
Multiple integration:	4065-4937-6534 bp	4061-6533-8134bp	3921-6114-6949 bp	4073-7763-9352 bp

Supplementary Fig. 6. Southern Blot analysis to confirm a single insertion event in SG200Δerc1 complementation. gDNA of both SG200 and complementation strains were digested with appropriate restriction enzyme. Cbx gene was used as a probe to detect single insertion event. Expected sizes for each insertion event depicted below figure.
