# Supplementary material for: A conserved enzyme of smut fungi facilitates cell-to-cell extension in the plant bundle sheath
Source: Nat Commun. 2022 Oct 12;13:6003. doi: 10.1038/s41467-022-33815-7 (PMC9556619; doi:10.1038/s41467-022-33815-7)
Supplement: Supplementary file 3 — Description of Additional Supplementary Files [file 41467_2022_33815_MOESM3_ESM.pdf]

Description of Additional Supplemetnary Files:

Supplementary Data 1: Contains all plasmids and oligomers used in this study.
