# Supplementary material for: A conserved enzyme of smut fungi facilitates cell-to-cell extension in the plant bundle sheath
Source: Nat Commun. 2022 Oct 12;13:6003. doi: 10.1038/s41467-022-33815-7 (PMC9556619; doi:10.1038/s41467-022-33815-7)
Supplement: Supplementary file 5 — Reporting Summary [file 41467_2022_33815_MOESM5_ESM.pdf]

nature portfolio

Corresponding author(s): Gunther Doehlemann, Bilal Ökmen

Last updated by author(s): September 30, 2022

Reporting Summary

Nature Portfolio wishes to improve the reproducibility of the work that we publish. This form provides structure for consistency and transparency in reporting. For further information on Nature Portfolio policies, see our Editorial Policies and the Editorial Policy Checklist.

Statistics

For all statistical analyses, confirm that the following items are present in the figure legend, table legend, main text, or Methods section.

n/a	Confirmed	
☐	☒	The exact sample size (*n*) for each experimental group/condition, given as a discrete number and unit of measurement
☐	☒	A statement on whether measurements were taken from distinct samples or whether the same sample was measured repeatedly
☐	☒	The statistical test(s) used AND whether they are one- or two-sided<br>*Only common tests should be described solely by name; describe more complex techniques in the Methods section.*
☐	☐	A description of all covariates tested
☐	☐	A description of any assumptions or corrections, such as tests of normality and adjustment for multiple comparisons
☐	☒	A full description of the statistical parameters including central tendency (e.g. means) or other basic estimates (e.g. regression coefficient) AND variation (e.g. standard deviation) or associated estimates of uncertainty (e.g. confidence intervals)
☐	☒	For null hypothesis testing, the test statistic (e.g. *F*, *t*, *r*) with confidence intervals, effect sizes, degrees of freedom and *P* value noted<br>*Give P values as exact values whenever suitable.*
☐	☒	For Bayesian analysis, information on the choice of priors and Markov chain Monte Carlo settings
☐	☐	For hierarchical and complex designs, identification of the appropriate level for tests and full reporting of outcomes
☒	☐	Estimates of effect sizes (e.g. Cohen's *d*, Pearson's *r*), indicating how they were calculated

Our web collection on statistics for biologists contains articles on many of the points above.

Software and code

Policy information about availability of computer code

Data collection	All softwares used in this study are decripted in the methods part including software version and references.
Data analysis	All softwares used in this study are decripted in the methods part including software version and references.

For manuscripts utilizing custom algorithms or software that are central to the research but not yet described in published literature, software must be made available to editors and reviewers. We strongly encourage code deposition in a community repository (e.g. GitHub). See the Nature Portfolio guidelines for submitting code & software for further information.

Data

Policy information about availability of data

All manuscripts must include a data availability statement. This statement should provide the following information, where applicable:

- Accession codes, unique identifiers, or web links for publicly available datasets
- A description of any restrictions on data availability
- For clinical datasets or third party data, please ensure that the statement adheres to our policy

The source data underlying Figs 1-6 and Supplementary Figs 1-6 are provided inthe source data file.
All data that support the findings of this study which are not directly available within the paper (and its supplementary information files) will be available from the corresponding authors (GD, BÖ) upon reasonable request.

Human research participants

Policy information about studies involving human research participants and Sex and Gender in Research.

Reporting on sex and gender	does not apply
Population characteristics	does not apply
Recruitment	does not apply
Ethics oversight	does not apply

Note that full information on the approval of the study protocol must also be provided in the manuscript.

Field-specific reporting

Please select the one below that is the best fit for your research. If you are not sure, read the appropriate sections before making your selection.

[x] Life sciences [ ] Behavioural & social sciences [ ] Ecological, evolutionary & environmental sciences

For a reference copy of the document with all sections, see nature.com/documents/nr-reporting-summary-flat.pdf

Life sciences study design

All studies must disclose on these points even when the disclosure is negative.

Sample size	All experiments were performed in three or more independent biological replicates to ensure statistical relevance and to exclude random effects appearing in experiments.
Data exclusions	Data of replicates were excluded when positive and/or negative controls did not show expected results. Without working controls experimental results cannot be stated to be true.
Replication	When technical problems were eliminated and handling of certain methods were established all experiments could be reliably reproduced. Of course, biological variations always occured within experiments and biological replicates.
Randomization	In this study, maize and barley plants were used as organisms. Plants were grown in standardized greenhouse conditions and randomly used for experiments.
Blinding	Blinding was not done in this study because chemicals and solutions had to be prepared prior to treatments/experiments with knowing what is in.

Reporting for specific materials, systems and methods

We require information from authors about some types of materials, experimental systems and methods used in many studies. Here, indicate whether each material, system or method listed is relevant to your study. If you are not sure if a list item applies to your research, read the appropriate section before selecting a response.

Materials & experimental systems

n/a	Involved in the study	
[ ]	[x]	Antibodies
[x]	[ ]	Eukaryotic cell lines
[x]	[ ]	Palaeontology and archaeology
[x]	[ ]	Animals and other organisms
[x]	[ ]	Clinical data
[x]	[ ]	Dual use research of concern

Methods

n/a	Involved in the study	
[x]	[ ]	ChIP-seq
[x]	[ ]	Flow cytometry
[x]	[ ]	MRI-based neuroimaging

Antibodies

Antibodies used	anti-HA antibody (Sigma Aldrich H-9658); goat anti-mouse antibody conjugated to 10 nm colloidal gold particles (British Biocell International, Cardiff, UK)
Validation	https://www.sigmaaldrich.com/deepweb/assets/sigmaaldrich/product/documents/391/208/h9658dat.pdf
